# Impact of Unilateral Selective Dorsal Rhizotomy (SDR) on Spasticity and Motor Function Improvement in Children With Hemiparetic Cerebral Palsy Caused by Intraventricular Hemorrhage

**DOI:** 10.7759/cureus.83641

**Published:** 2025-05-07

**Authors:** Sevgi Sarikaya-Seiwert, Ralf Clauberg, Ina Hainmann, Hartmut Vatter, Hannes Haberl, Ehab Shabo

**Affiliations:** 1 Section of Pediatric Neurosurgery, Department of Neurosurgery, University Hospital Bonn, Bonn, DEU; 2 Department of Neuroradiology, University Hospital Bonn, Bonn, DEU; 3 Department of Pediatric Hematology and Oncology, University Hospital Bonn, Bonn, DEU; 4 Department of Neurosurgery, University Hospital Bonn, Bonn, DEU

**Keywords:** cerebral palsy, hemiparesis, intraventricular hemorrhage, motor function, selective dorsal rhizotomy, spasticity, unilateral

## Abstract

Background

Selective dorsal rhizotomy (SDR) is an established surgical treatment for reducing spasticity in children with cerebral palsy (CP). However, its role in cases of hemiparetic spastic CP due to intraventricular hemorrhage (IVH) remains less well defined, especially regarding the potential benefit of unilateral SDR. Moreover, the majority of existing studies primarily focus on the effectiveness of SDR in reducing spasticity based on quantitative scoring systems, with no evaluation of clinically meaningful improvements in motor function. This study investigates the clinical outcomes of unilateral SDR in children with spastic hemiparetic CP resulting from IVH, with a particular focus on both the reduction of spasticity and improvements in motor function at 6 and 12 months following surgery.

Materials and methods

This retrospective monocentric study included 11 pediatric patients with IVH-induced hemiparetic CP who underwent a unilateral SDR between 2017 and 2023. IVH was classified using Volpe’s grading system. Pre- and postoperative assessments included the Gross Motor Function Classification System (GMFCS), Modified Ashworth Scale (MAS), as well as functional motor domains (sitting, standing, and walking) at 6 and 12 months after surgery. Furthermore, an additional analysis was conducted to assess the relationship between IVH severity and postoperative outcomes.

Results

All patients demonstrated complete resolution of spasticity following surgery, with postoperative MAS scores reduced to 0. In contrast, GMFCS levels remained unchanged in most patients, with improvement observed in only one case. Remarkably, all patients demonstrated improvements in functional motor skills at both the 6- and 12-month follow-ups, with no evidence of regression or recurrence of motor disability. No adverse events were observed. Notably, higher grades of IVH were not significantly associated with presurgical MAS scores (p=1.0) or GMFCS levels (p=0.45), and no meaningful correlation was observed between IVH severity and postoperative clinical outcomes.

Conclusion

Unilateral SDR is a promising and safe approach for children with hemisymptomatic CP resulting from IVH, leading to significant reductions in spasticity and sustained functional motor gains regardless of IVH severity. Further and larger prospective studies are needed to validate these findings.

## Introduction

Cerebral palsy (CP) is a prevalent neurodevelopmental disorder resulting from non-progressive brain injury during early development, leading to motor impairments and spasticity [1]. Spastic hemiplegia, a subtype of cerebral palsy, is characterized by unilateral hypertonia. Intraventricular hemorrhage (IVH), a frequent complication of prematurity, is recognized as the primary and most common etiological factor for cerebral palsy in preterm infants, as hemorrhagic injury to the periventricular white matter disrupts the developing corticospinal tracts and leads to significant motor impairments, particularly unilateral deficits characteristic of spastic hemiplegia [2-5].

Selective dorsal Rhizotomy (SDR) has been established as an effective surgical intervention for reducing spasticity in children with spastic diplegia, demonstrating long-term improvements in muscle tone and motor function [6]. However, the role of SDR in children with hemisymptomatic spastic CP, particularly those resulting from IVH, remains not explored. In such cases, a unilateral approach to SDR may offer a more targeted intervention with potentially fewer side effects and challenging recovery times compared to bilateral SDR [7].

While many of the existing studies report significant reduction in spasticity, they primarily focus on quantitative measures and scoring systems such as GMFCS and AMS without thoroughly assessing clinically meaningful improvements in motor function [8-15].

Postoperative impairments in coordination and leg function during the initial weeks following SDR are well-known. In cases of bilateral SDR, both legs are similarly affected, whereas unilateral SDR spares the non-operated side from these temporary functional limitations. However, the operated side often experiences significant restrictions. This asymmetrical impairment can hinder effective rehabilitation during the first 3 to 6 weeks after surgery.

This study aims to evaluate the neurological outcomes of unilateral SDR in a cohort of pediatric patients with hemiparetic CP caused by IVH, focusing on spasticity, measured by Modified Ashworth Scale (MAS) scores, Gross Motor Function Classification System (GMFCS), as well as fundamental motor skills (sitting, standing and walking) over a 12-month postoperative period.

## Materials and methods

Patients’ selection and inclusion criteria

In this retrospective study, we included all patients who underwent a unilateral SDR for treatment of hemiparetic spastic CP secondary to IVH at our institute between 2017 and 2023.

In accordance with Volpe’s grading system, IVH was classified into four categories [16-18]: Grade I refers to subependymal hemorrhage into the germinal matrix without IVH or with IVH involving less than 10% of the intraventricular volume. Grade II describes IVH that involves 10-50% of the ventricular volume. Grade III is characterized by IVH that involves more than 50% of the ventricular volume, potentially accompanied by ventricular dilatation. Periventricular venous hemorrhagic infarction (PVHI) is considered a separate entity, as it results from, but does not extend from IVH.

Surgical approach - unilateral SDR

At our institution, the indication for SDR is established through interdisciplinary assessment and tailored according to the clinical subtype of CP. For patients with spastic diplegia or quadriplegia, bilateral SDR is routinely performed. In contrast, individuals with well-defined hemispastic CP undergo unilateral SDR limited to the affected side, with preservation of the contralateral, unaffected rootlets. This selective approach reduces operative time while maintaining comparable clinical outcomes.

In this study, all patients underwent unilateral SDR, targeting the lumbosacral dorsal rootlets on the side corresponding to the hemiparetic presentation. Intraoperative neurophysiological monitoring was used to identify and selectively section the hyperactive rootlets, while preserving functional motor responses. The contralateral side was not addressed surgically.

GMFCS and MAS were used to classify gross motor function and assess spasticity, respectively, before and after the SDR procedure.

Specific functional abilities were evaluated using a two-step approach. First, a visual gait assessment scale was applied [19], followed by a clinical evaluation of three key motor function skills: standing, sitting, and walking. Functional outcomes were categorized as “improved,” “unchanged,” or “worsened” based on both approaches. These assessments were conducted during follow-up visits at 6 and 12 months postoperatively in the outpatient clinic.

The clinical evaluation of movement disorders in all patients was conducted at the time of first diagnosis as well as during follow-ups in an interdisciplinary setting involving neurosurgeons, orthopaedic surgeons, pediatric neurologists, and physiotherapists.

Statistical analysis

Patient demographics, IVH grade, MAS and GMFCS scores, and functional outcomes were recorded in a structured database. Descriptive statistics were used to summarize the findings. All data analysis was performed using IBM® SPSS® Statistics (version 27, IBM Corp., Armonk, NY). Nominal data were tested between groups with small counts using Fisher’s exact test. Independent t-test was used to compare means between two independent groups. A p-value <0.05 was considered statistically significant. 

## Results

Patient cohort and general characteristics

A total of 11 patients (eight males, three females) met the inclusion criteria and were included in the final analysis. Nine patients were born preterm (81.8%), while the remaining two were born at term.

The average age at SDR was 35.3 months (range: 27-42 months).

Among the study cohort, no patients were classified with IVH Grade I. Three patients (27.3%) had Grade II IVH, and another three patients (27.3%) had Grade III IVH. The remaining five patients (45.4%) were diagnosed with periventricular venous hemorrhagic infarction (PVHI).

Patients’ characteristics are summarized in Table 1.

**Table 1:** Detailed Patients’ Characteristics. GMFCS: Gross Motor Function Classification System; IVH: Intraventricular Hemorrhage; MAS: Modified Ashworth Scale; No.: Number; SDR: selective dorsal rhizotomy; PVHI: Periventricular venous hemorrhagic infarction

No. of Patients			11
Male			8 (72.7%)
Preterm			9 (81.8%)
IVH Grade	I		0
	II		3 (27.3%)
	III		3 (27.3%)
	PVHI		5 (45.4%)
Age at SDR (months)			35.3 (range: 27-42)
GMFCS pre-SDR		level II	2 (18.2%)
		level III	8 (72.7%)
		level IV	1 (9.1%)
GMFCS post-SDR		level II	3 (27.3%)
		level III	7 (63.6%)
		level IV	1 (9.1%)
MAS Score pre-SDR		3	3 (27.3%)
		4	8 (72.7%)
MAS Score post-SDR: 0			11 (100%)
Follow-up at 6 months (standing, sitting, walking)			all improved (100%)
Follow-up at 12 months (standing, sitting, walking)			all improved (100%)

Development of spasticity and motor function after SDR

Prior to SDR, the majority of patients (72.7%) were classified as GMFCS level III. Post-SDR, the GMFCS level remained unchanged in 10 patients, while only one patient experienced a one-level improvement, suggesting that SDR did not result in significant overall improvements in mobility.

Regarding spasticity, the MAS score before SDR indicated that eight patients (72.7%) had a score of 4, and the remaining three (27.3%) had a score of 3. After SDR, all patients exhibited a MAS score of 0, demonstrating a successful reduction of spasticity.

In terms of functional outcomes, all patients (100%) showed clinical improvement in motor skills (sitting, standing, and walking) according to the visual gait assessment scale at both 6 and 12 months follow-up. These improvements were consistent and sustained across both time points, indicating a lasting benefit of the procedure on meaningful motor abilities.

Neuroradiological correlation with patients’ characteristics and pre-surgical motor function

A subsequent statistical comparison between patients with IVH (Grades II and III) and those with PVHI revealed several key findings. Birth type distribution (preterm vs. term), sex (male v. female), and the average age at SDR were similar across both groups, with no significant difference (all p-values were above 0.05 as demonstrated in Table 2).

**Table 2:** Statistically compares IVH and PVHI regarding patients’ characteristics. GMFCS: Gross Motor Function Classification System; IVH: Intraventricular Hemorrhage; MAS: Modified Ashworth Scale; No.: Number; SDR: selective dorsal rhizotomy; PVHI: Periventricular venous hemorrhagic infarction.

		IVH (Grade II or III)	PVHI	p-Values	Statistical test
Patients No. (%)		6 (54.5%)	5 (45.4%)		
Birth	preterm	4 (66.6%)	5 (100%)	0.45	Fisher’s exact test
	term	2 (33.3%)	0		
Sex	male	5 (83.3%)	3 (60%)	0.54	Fisher’s exact test
	female	1 (16.7%)	2 (40%)		
Age at SDR (months)		35.0 (Range: 27- 41)	35.6 (Range: 29-42)	0.52	Independent t-test (t value: -0.7)
Pre-SDR GMFCS	level II	2 (33.3%)	0	0.45	Fisher’s exact test
	level III or IV	4 (66.6%)	5 (100%)		
Pre-SDR MAS	3	2 (33.3%)	1 (20%)	1	Fisher’s exact test
	4	4 (66.6%)	4 (80%)		

Regarding motor function, pre-SDR GMFCS levels were categorized into two groups (level II vs. levels III or IV). No significant differences were observed between the severity of IVH and pre-SDR GMFCS levels (p = 0.45) or spasticity, as measured by MAS score (p = 1). These findings suggest that the severity of IVH does not correlate with the degree of motor function deficits or clinical presentation.

The statistical analysis is presented in Table 2. Details about patients’ characteristics and motor function development are presented in Table 3.

**Table 3:** Detailed Patients’ Characteristics. GMFCS: Gross Motor Function Classification System; IVH: Intraventricular Hemorrhage; MAS: Modified Ashworth Scale; No.: Number; SDR: selective dorsal rhizotomy; PVHI: Periventricular venous hemorrhagic infarction

Patient No.	Birth	Sex	IVH Grade	PVHI extent	Age at SDR (months)	GMFCS		MAS score		Follow-up after 6 months (visual gait assessment scale)			Follow-up after 12 months (visual gait assessment scale)		
						pre- SDR	post- SDR	pre-SDR	post-SDR	standing	sitting	walking	standing	sitting	walking
1	preterm	male	II	n.d.	27	III	II	4	0	improved	improved	improved	improved	improved	improved
2	preterm	male	PVHI	>2 cm	31	III	III	4	0	improved	improved	improved	improved	improved	improved
3	preterm	male	III	n.d.	33	III	III	4	0	improved	improved	improved	improved	improved	improved
4	preterm	male	PVHI	>2 cm	40	III	III	3	0	improved	improved	improved	improved	improved	improved
5	term	female	II	n.d.	35	III	III	3	0	improved	improved	improved	improved	improved	improved
6	preterm	female	PVHI	1-2 cm	29	III	III	4	0	improved	improved	improved	improved	improved	improved
7	preterm	male	PVHI	>2 cm	42	IV	IV	4	0	improved	improved	improved	improved	improved	improved
8	term	male	III	n.d.	39	III	III	4	0	improved	improved	improved	improved	improved	improved
9	preterm	male	II	n.d.	35	II	II	3	0	improved	improved	improved	improved	improved	improved
10	preterm	female	PVHI	1-2 cm	36	III	III	4	0	improved	improved	improved	improved	improved	improved
11	preterm	male	III	n.d.	41	II	II	4	0	improved	improved	improved	improved	improved	improved

## Discussion

Unilateral SDR has emerged as a promising intervention for children with hemiparetic spastic CP, providing a more focused neurosurgical approach compared to the traditional bilateral SDR. This method targets the affected side selectively, aiming to reduce spasticity while preserving function in the less affected limb. Prior studies have suggested that unilateral SDR may offer advantages such as reduced operative duration, fewer complications, and a shorter recovery period [7]. Despite these benefits, the application of unilateral SDR in children with hemisymptomatic CP specifically resulting from IVH, the most common pathophysiological cause of CP in infants, remains understudied.

Previous research has established the efficacy of SDR in reducing lower limb spasticity and improving gross motor function in spastic diplegia and quadriplegia, with most outcomes assessed using standardized scales such as GMFCS and MAS [8-15]. However, many of these studies have focused on broader CP populations, often without stratifying by etiology or lesion type, such as IVH or PVHI. As a result, the unique responses of children with hemisymptomatic CP due to IVH to unilateral SDR remain poorly characterized in the literature.

Our study contributes to filling this gap by specifically evaluating children with hemiparetic CP secondary to its most common cause (IVH and PVHI), assessing not only spasticity and GMFCS levels but also more nuanced clinical markers of motor function, including sitting, standing, and walking performance over a 12-month postoperative period.

The complete postoperative resolution of spasticity, as evidenced by the decrease in MAS scores to 0 in all patients, is consistent with the robust spasticity reduction reported in earlier studies that explored SDR in diplegic and hemiplegic presentations [6, 7]. Notably, our findings support and extend these prior observations by demonstrating that this reduction is achievable even in patients with complex neuropathological findings such as PVHI, who are often considered at higher risk for poor outcomes.

Moreover, while the GMFCS level remained unchanged in most patients, mirroring findings from prior studies indicating that GMFCS may not be sensitive enough to capture subtle motor gains post-SDR [20], the significant improvements in specific motor functions (i.e., sitting, standing, and walking) highlight the clinical relevance of using complementary functional assessments such as the visual gait assessment scale, therefore shifting from purely classification-based metrics to more meaningful, patient-centred functional outcomes.

Notably, no correlation was found between radiological severity of IVH and preoperative spasticity, measured by MAS score, or gross motor function, measured by GMFCS levels, suggesting that the extent of parenchymal injury may not directly reflect baseline motor impairment. Furthermore, the severity of IVH did not predict poorer surgical outcomes. All patients, irrespective of initial radiological severity or clinical presentation, experienced substantial reductions in spasticity and meaningful improvements in functional mobility following SDR. These findings underscore the effectiveness of SDR across a spectrum of initial radiological and clinical severities and highlight its potential benefit even in cases with extensive brain injury.

Our results also offer practical clinical value. The fact that children with higher-grade IVH or PVHI responded similarly well to SDR may provide reassurance to clinicians and families facing early prognostic uncertainty following neonatal brain injury. Given that IVH grading can be distressing during the neonatal period, our data suggest that early severity may not be a definitive predictor of long-term functional limitations if appropriate interventions like SDR are employed.

These findings add to the growing body of evidence advocating for tailored approaches in CP management and support the continued exploration of unilateral SDR as a viable option in select patient populations.

Overall, this study represents the first report in the literature specifically addressing this distinct subgroup of CP patients with a history of IVH. The key finding of this study (namely, the absence of a correlation between IVH grade and clinical outcomes) may, if validated by larger prospective studies, challenge current perceptions of the prognostic relevance of higher-grade IVH and provide increased reassurance to patients and families facing severe IVH.

Nonetheless, our study is limited by its small sample size and retrospective design, which restricts statistical power and generalizability. The lack of a control group is also an additional limitation. Larger, multicenter prospective studies are needed to confirm these findings, better delineate the relationship between IVH grade and postoperative improvement, and assess the durability of these outcomes into adolescence and adulthood.

## Conclusions

This study highlights the potential of unilateral SDR as an effective surgical option for reducing spasticity and improving functional mobility in children with hemiparetic CP secondary to IVH or PVHI. By targeting the affected side, unilateral SDR offers a focused approach that may preserve motor function in the less involved limbs while still delivering meaningful improvements in daily activities such as sitting, standing, and walking. Importantly, our findings also suggest that the severity of early brain injury does not necessarily limit the potential for functional gains following SDR, offering reassurance for clinical decision-making in cases with complex neuroimaging findings. Although traditional classification systems may not fully reflect postoperative progress, individualized functional assessments can reveal significant benefits. These insights support a more nuanced and patient-centered approach to surgical intervention in this vulnerable population.
